# Mapping pharmacy deserts and determining accessibility to community pharmacy services for elderly enrolled in a State Pharmaceutical Assistance Program

**DOI:** 10.1371/journal.pone.0198173

**Published:** 2018-06-04

**Authors:** Priti Pednekar, Andrew Peterson

**Affiliations:** Department of Health Policy, University of the Sciences, Philadelphia, Pennsylvania, United States of America; Stony Brook University, Graduate Program in Public Health, UNITED STATES

## Abstract

**Objectives:**

Limited studies have investigated geographic accessibility to a nearby community pharmacy for elderly which is an essential determinant of the access to medications and pharmacy services. This research identified pharmacy deserts and investigated availability of different types of community pharmacies and their services for elderly enrolled in a State Pharmaceutical Assistance Program (SPAP).

**Methods:**

The state of Pennsylvania in the US was used as a case to demonstrate the geographic accessibility to community pharmacy and services for elderly enrolled in SPAP. The locations of community pharmacies and households of elderly enrolled in SPAP were derived from Pharmaceutical Assistance Contract for the Elderly programs’ database. The street addresses were geocoded and the distance to a nearby community pharmacy was calculated for study sample using the haversine formula. The demographic and geographic data were aggregated to Census Tracts and pharmacy deserts were identified using the predefined criteria. Descriptive statistical analysis was used to determine whether there are statistical differences in the socio-demographic profiles and distribution of different types of community pharmacies and their services in pharmacy deserts and non-deserts. This research used hot spot analyses at county level to identify clusters of pharmacy deserts, areas with high concentration of different racial/ethnic groups and clusters of high densities of chain and independent pharmacies.

**Results:**

The Spatial analysis revealed that 39% and 61% Census Tracts in Pennsylvania were pharmacy deserts and non-deserts respectively (p < 0.001). Pharmacy deserts were found to have significantly more females, married and white elderly and fewer blacks and Hispanics compared to pharmacy non-deserts. Pharmacy deserts had significantly fewer chain and independent pharmacies and less delivery and 24-hour services in pharmacies than pharmacy non-deserts. Hot spot analyses showed that clusters of pharmacy deserts were more concentrated in southcentral, northwest and northeast regions of the state which represent rural areas and overlapped with clusters of high concentration of white individuals.

**Conclusions:**

The findings suggest that urban-rural inequality, racial/ethnic disparity and differences in availability of pharmacies and their services exist between pharmacy deserts and non-deserts. The methodological approach and analyses used in this study can also be applied to other public health programs to evaluate the coverage and breadth of public health services.

## Introduction

Elderly use prescription medications more than any other age groups as they are more likely to have multiple and/or severe chronic conditions and more frequent seasonal illnesses[1,2]. In the US, elderly use about 25–30% of all prescription medications[3]. An elderly patient takes, on average, four or five prescription drugs and two over-the-counter (OTC) medications[4]. Elderly people are also more likely to have adverse drug reactions, drug-drug interactions and medication errors[5–7]. The accessibility to a pharmacy is therefore an important facilitator of overall health of elderly population.

In addition to dispensing medications, the community pharmacies offer a wide spectrum of services including patient counselling, screening tests, immunization services, wellness programs, and education programs[8]. With extended hours of operation, availability of home delivery of medications, and no need to schedule an appointment for counselling, community pharmacies are in the unique position being the most accessible than the other healthcare settings. Previous studies have indicated that the pharmacists, in collaboration with physicians and other health professionals, can improve medication safety[9], promote health, prevent disease, improve medication adherence[10,11] and other patient outcomes[12] and reduce health care costs[13]. For example, the group of patients which received medication management interventions by a community pharmacy had 3% higher medication adherence, 1.8% less hospital admissions, 2.7% less emergency room visits, and 0.53 fewer mean outpatient visits compared to the group of patients which did not receive medication management services from a community pharmacy[14]. Because of this role, the accessibility to community pharmacies is critical to ensure proper utilization of medications and adequate delivery of healthcare services.

Previous studies investigating determinants of access to prescription medications for elderly focused on physical and psychological patient factors and socio-economic factors such as income, cost of medication, lack of health insurance and prescription drug coverage[15–17]. In addition to economic factors, geographic accessibility to a community pharmacy is also an essential determinant of the access to prescription medications as it may affect the older individual’s ability to fill prescriptions even in the absence of economic barriers. There are several State Pharmaceutical Assistance Programs (SPAPs) for older adults which provide free or low-cost prescription drugs to low-income older individuals[18]. These programs however, require older individuals to fill their prescriptions in pharmacies which participate in SPAPs. Elderly people that live in communities without such pharmacy, or require travelling more to find such pharmacy, may experience geographic barriers to fill their prescriptions regardless of their financial access. Recently, the term ‘pharmacy deserts’ was coined based on the concept of ‘food deserts’ and it refers to geographic areas which lack access to a nearby pharmacy and where pharmacy services are scarce or difficult to obtain[19]. There is a lack of knowledge about the pharmacy deserts for an SPAP-enrolled elderly population. It is important to investigate because elderly living in pharmacy deserts may experience greater difficulty in accessing medications and other pharmacy services, even if economic access to medications was improved through SPAPs. It may further result in increased medication non-adherence and ultimately poorer health outcomes and increased healthcare cost.

Spatial analytical methods and Geographic Information Systems (GIS) have been used to assess the accessibility to pharmacies and mainly focused on the racial/ethnic disparity or urban-rural inequality [19–26]. In Chicago, pharmacy deserts were predominantly present in segregated African-American and Hispanic communities as compared to segregated white and integrated communities. The types of pharmacies available also significantly differed across communities in 2012; white communities had more chain (58.6% vs. 38%) and less independent (23.3% vs. 38%) pharmacies than black communities[19]. In New York City health districts, Cooper et al. also found that white residents had substantially greater geographical access to pharmacies than black residents[26]. However, Ikram examined accessibility to pharmacies in Baton Rouge, Louisiana and reported that more African-Americans were in areas closer to pharmacies, in terms of travel time, as compared to Whites, but these areas had fewer pharmacies per 10,000 residents[25]. In 2004, Lin compared the geographic accessibility to retail pharmacies for elderly between rural and urban areas in Illinois. This study found that 93.8% of pharmacies were located in urban areas. The mean number of pharmacies per 10,000 elderly patients was less in rural areas compared to urban areas. Lin also calculated the travel distance to nearest pharmacy and found that it was less in urban areas (0.9 miles) than rural areas (5.9 miles)[24].

Several key concerns can be raised about this earlier research. These studies identified geographic accessibility to pharmacy based on the ‘centroid approach’ which considers the simple geographic centroid of a geospatial unit of analysis (for example, census tracts, Zip codes or census blocks) as the location of the population and measures the straight-line distance between the centroid and its corresponding nearest facility. Though the ‘centroid approach’ is commonly used by researchers in locational analysis, it uses aggregated data and does not calculate the distance between the household address of each individual in the population and the closest pharmacy. This may introduce an error because it is possible that the actual distance that an individual needs to travel to visit a pharmacy is less than the distance between the centroid and its corresponding nearest pharmacy. Several studies have used density of pharmacies or population density per pharmacy to examine spatial access to pharmacies[20,21,27]. However, these measures do not truly measure geographical access to pharmacies. Several studies measured access as a driving distance. Since driving retirement is inevitable for most elderly individuals, such measurement methods may not be the best way to evaluate access to pharmacies[19,28,29]. Our study, however, identified pharmacy deserts using geospatial methods based on the distance between the household address and nearest community pharmacy.

Hot spot analysis is a statistically based method used to identify spatial clusters of high and low values of a phenomenon of interest. Recent studies using this technique have studied geographic variations in medication adherence[30,31], health care spending[32], locations of over-the-counter (OTC) syringe-selling pharmacies[33], health disparities in delivery of primary care[34], availability of healthcare professionals[35] and locations requiring more Women, Infants, and Children (WIC) program services[36]. To our knowledge, however, hot spot analysis has not been employed to identify clusters of pharmacy deserts for elderly enrolled in SPAPs.

The objective of our study was to identify pharmacy deserts in Pennsylvania for elderly enrolled in the SPAP. Data were mapped and distances were calculated between the household address and nearest community pharmacy participating in the SPAP. The study identified the county-level geographic regions of Pennsylvania that contain clusters of pharmacy deserts. Additional analysis examined urban-rural inequalities, racial/ethnic disparity and geographic variation in distribution of different types of community pharmacies and availability of their services in pharmacy deserts and non-deserts. Findings were intended to provide the Pennsylvania’s SPAP with an evidence base for funds allocation, to inform program decisions for a subset of counties and census tracts, and to strategically target specific areas and demographic groups which experience poorer access to community pharmacies.

## Materials and methods

### Study area and data sources

This study focuses on the state of Pennsylvania, which is a single state in the US that offers two pharmaceutical assistance programs for elderly[37]. For this study, Pennsylvania was divided into six geographic regions (Table 1). The U.S 2010 Census definitions for Urban and rural areas at county level were used for this study.

**Table 1:** Geographic regions and counties in Pennsylvania.

Geographic Region	County
*Northeast*	Bradford, Lackawanna, Luzerne, Lycoming, Pike, Sullivan, Susquehanna, Tioga, Wayne, Wyoming
*Central*	Cameron, Centre, Clearfield, Clinton, Potter, Union
*Northwest*	Clarion, Crawford, Elk, Erie, Forest, Jefferson, McKean, Mercer, Venango, Warren
*Southwest*	Allegheny, Armstrong, Beaver, Butler, Fayette, Greene, Indiana, Lawrence, Washington, Westmoreland
*Southcentral*	Bedford, Blair, Cambria, Franklin, Fulton, Huntingdon, Juniata, Mifflin, Northumberland, Perry, Snyder, Somerset
*Southeast*	Adams, Berks, Bucks, Carbon, Chester, Columbia, Cumberland, Dauphin, Delaware, Lancaster, Lebanon, Lehigh, Monroe, Montgomery, Montour, Northampton, Philadelphia, Schuylkill, York

This cross-sectional study used data obtained from Pennsylvania’s Pharmaceutical Assistance Contract for the Elderly (PACE) and the PACE Needs Enhancement Tier (PACENET) programs’ database (together referred to as PACE dataset). The PACE and PACENET are state-funded pharmaceutical assistance programs administered by the Pennsylvania Department of Aging that offer low-cost prescription medications to more than 300,000 older adults with limited incomes[37]. A data use agreement was signed by study investigators prior to obtaining the data. The study was approved by the University of the Sciences’ Institutional Review Board.

PACE dataset provided demographic characteristics of the study subjects such as age, sex, annual household income, marital status, race and ethnicity, along with the street address where the patient lived at the time of enrollment. Data on community pharmacies (pharmacies licensed to dispense prescriptions to the public in Pennsylvania) were obtained from PACE and included the name and type of pharmacy as well as street address of each pharmacy. These data also provided information about services offered by pharmacy such as delivery services, emergency delivery services, times and days of operation.

### Identification of pharmacy deserts

#### Step 1: Mapping community pharmacies

The list of pharmacies which were licensed to practice in Pennsylvania and participated in PACE program in 2015 was acquired from PACE. We included independently pharmacist-owned pharmacies and pharmacies attached to a corporate chain store. They were labeled as “independent pharmacies” and “chain pharmacies” respectively; and together were referred as “community pharmacies”. As we were interested in geographic accessibility to community pharmacies which serve non-institutionalized walk-in elderly individuals, we excluded pharmacies located in nursing homes and hospitals, home infusion pharmacies, pharmacies that fill prescriptions over internet and mail-order pharmacies.

The dataset obtained from PACE included the street address of community pharmacies, a composite of house number, street name, city, state and zip code. The street addresses of community pharmacies included in the study were geocoded into geographic co-ordinates (longitude and latitude) using ArcGIS 10.5 software (ESRI Redlands, CA). We observed 100% geocoding matching rate. We then conducted a “point in polygon join” (i.e., reverse geocoding) within the ArcGIS to assign a US 2010 census tract number to each geocoded address point. Total 2,752 eligible community pharmacies were mapped, of which 36% were independent pharmacies and remaining 64% were chain pharmacies.

#### Step 2: Mapping households of PACE enrollees

Elderly residents of Pennsylvania who were alive and continuously enrolled in the PACE program for a full year starting January 1, 2015 were included in the study regardless of their disease status. For this study, these subjects were referred to as ‘enrollees’. Enrollees must have been at least 65 years of age at the time of enrollment to be included in the study. Enrollees who were institutionalized (e.g., living in nursing homes or long-term care settings) were excluded because our study intended to calculate the distance between enrollee’s home and nearest community pharmacy. Total 216,350 PACE enrollees were found to be eligible.

The dataset obtained from PACE included the street address of households of enrollees included in the study, a composite of house number, street name, city, state and zip code. The street addresses of households were geocoded into geographic co-ordinates (longitude and latitude) using ArcGIS 10.5 software (ESRI Redlands, CA). Out of eligible PACE enrollees (n = 216,350), 43,383 patients were not geocoded because of missing street addresses. The geocoding mapped households of total 172,967 PACE enrollees (geocoding accuracy rate = 75%). We then conducted a “point in polygon join” (i.e., reverse geocoding) within the ArcGIS to assign a US 2010 census tract number to each geocoded address point.

#### Step 3: Distance to a nearest community pharmacy

The distance between household of enrollee and each community pharmacy (independent or chain pharmacy) in Pennsylvania were calculated (in miles) using geographic coordinates. These distances were computed for each enrollee using the ‘haversine’ formula with SAS software, v.9.4 (SAS Institute, Inc., Cary, NC)[38]:

d=R*arcos(sin(lat1)*sin(lat2)+cos(lat1)*cos(lat2)*cos(long2−long1)), (1)

where:

d = Distance from a household of an enrollee to a community pharmacy in miles

R = Polar Radius of earth in miles (3949.99 miles)

Lat1 = Latitude of enrollee’s household location in radians

Long1 = Longitude of enrollee’s household location in radians

Lat2 = Latitude of community pharmacy’s location in radians

Long2 = Longitude of community pharmacy’s location in radians

The shortest distance (in miles) was considered as the distance required for an enrollee to visit a nearest community pharmacy. The haversine formula considers spherical shape of the earth and is appropriate when two places on the earth are close to each other[39–45]. This study did not use ‘centroid approach’ as it does not precisely measure proximity. We considered the fact that enrollees may visit pharmacies in neighboring census tracts within Pennsylvania, however the community boundaries were restricted to the state of Pennsylvania only. Hence, nearest community pharmacy identified can be in the same or neighboring census tract in Pennsylvania.

#### Step 4: Identifying pharmacy deserts

Details of sociodemographic characteristics and distance to a nearby community pharmacy for enrollees were aggregated at Census Tract level. A census tract was designated as a ‘pharmacy desert’ if it had more than 33% of enrollees living more than 1 mile from a nearby community pharmacy [19]. Census tracts which had households of enrollees but were not considered as pharmacy deserts according to the aforementioned criteria, were denoted as ‘pharmacy non-deserts’. For the purpose of this study, we opted to define ‘pharmacy deserts’ at U.S. Census 2010 tracts level because they cover well-defined small geographic area, provide more granularity and have definite boundaries[27,46]. In addition, the concept of ‘pharmacy deserts’ is based on the ‘food deserts’ which have been defined at census tract level by the US Department of Agriculture and the Centers for Disease Control and Prevention[47,48].

At county level, proportion and percentage of pharmacy deserts were calculated:

$$\text{Proportion}\;\text{of}\;\text{Pharmacy}\;\text{Deserts}\;\left(\text{PPD}\right)=\frac{\text{Number}\;\text{of}\;\text{census}\;\text{tracts}\;\text{identified}\;\text{as}\;\text{pharmacy}\;\text{deserts}\;\text{in}\;\text{a}\;\text{county}}{\text{Total}\;\text{number}\;\text{of}\;\text{census}\;\text{tracts}\;\text{present}\;\text{in}\;\text{a}\;\text{county}}$$ (2)

Percentageofpharmacydeserts=PPD*100, (3)

### Statistical analysis

Basic descriptive statistics for sociodemographic characteristics of enrollees were estimated. Population density was calculated for pharmacy deserts and pharmacy non-deserts by dividing the number of enrollees residing in those areas by respective land areas in square miles. For comparing socioeconomic and demographic characteristics between enrollees residing in pharmacy deserts and pharmacy non-deserts, t-tests for continuous variables and Chi-square tests for categorical variables were used. Total pharmacy density was computed per 100 enrollees for pharmacy deserts and pharmacy non-deserts by dividing total number of pharmacies in the community (pharmacy deserts or pharmacy non-deserts) by the total number of enrollees living in the community. Similarly, the density of independent and chain pharmacies per 100 enrollees was computed for pharmacy deserts and pharmacy non-deserts. The pharmacy densities and frequency distribution of services offered by community pharmacies in pharmacy deserts and pharmacy non-deserts were compared using t-test and chi-square test. All statistical analyses were performed using SAS 9.4 software (Cary, NC). All comparisons had statistical significance at levels < 0.05.

We used descriptive GIS mapping techniques to assess the spatial distribution of pharmacy deserts at the state and county levels. We explored the density of PACE enrollees per square mile and percentages of pharmacy deserts at county level with thematic maps. Proportion of Pharmacy Deserts, percentages of PACE enrollees by racial and ethnic groups and densities of independent and chain pharmacies per 100 PACE enrollees were computed at county level.

To determine the location of statistically significant clusters of pharmacy deserts at county level, hot spot analysis was conducted using ArcGIS 10.5 software (ESRI Redlands, CA). This spatial analysis calculates Getis-ORD Gi* statistic for each feature (e.g., county), within the context of neighboring features and against all features in the dataset, and results in a z-score and p-value. From this, one can determine an area of high or low occurrence and if that result is too great to be due to chance. A small negative z-score and a small p-value indicates a statistically significant low-value clusters (cold spots) of a phenomenon of interest. The local mean for a feature along with its neighbors is compared with the mean of all features (e.g., all counties in a state). When the local mean is much different than the expected global mean and the magnitude of difference is too great to be due to chance, a statistically significant *z*-score results that indicates a hot spot cluster[49].

In the current study, the hot spot analysis tool calculated Getis-ORD Gi* statistic for each county and resulted in a z-score and p value. The larger the z-score, the more concentrated the clustering of high values (hot spot). The lower the z-score, the more concentrated the clustering of low values (cold spot). While determining the statistically significant clusters of pharmacy deserts, hot spots refer to higher PPD, while cold spots refer to lower PPD. A county with a z-score that is neither high nor low is considered neutral and it was not statistically significantly different from its neighbors. Similarly, hot spot analysis was conducted to determine the statistically significant clusters of higher densities of chain and independent pharmacies in Pennsylvania. Hot spots refer to counties with higher densities of chain and independent pharmacies and cold spots refer to lower densities of chain and independent pharmacies. Hot spot analysis was also conducted to determine the locations of statistically significant clusters of enrollees of specific racial and ethnic group, where hot spots and cold spots refer to higher and lower percentage of enrollees with particular race or ethnicity respectively.

## Results

### I. Spatial distribution and hot spot analysis of pharmacy deserts

**Fig 1:**
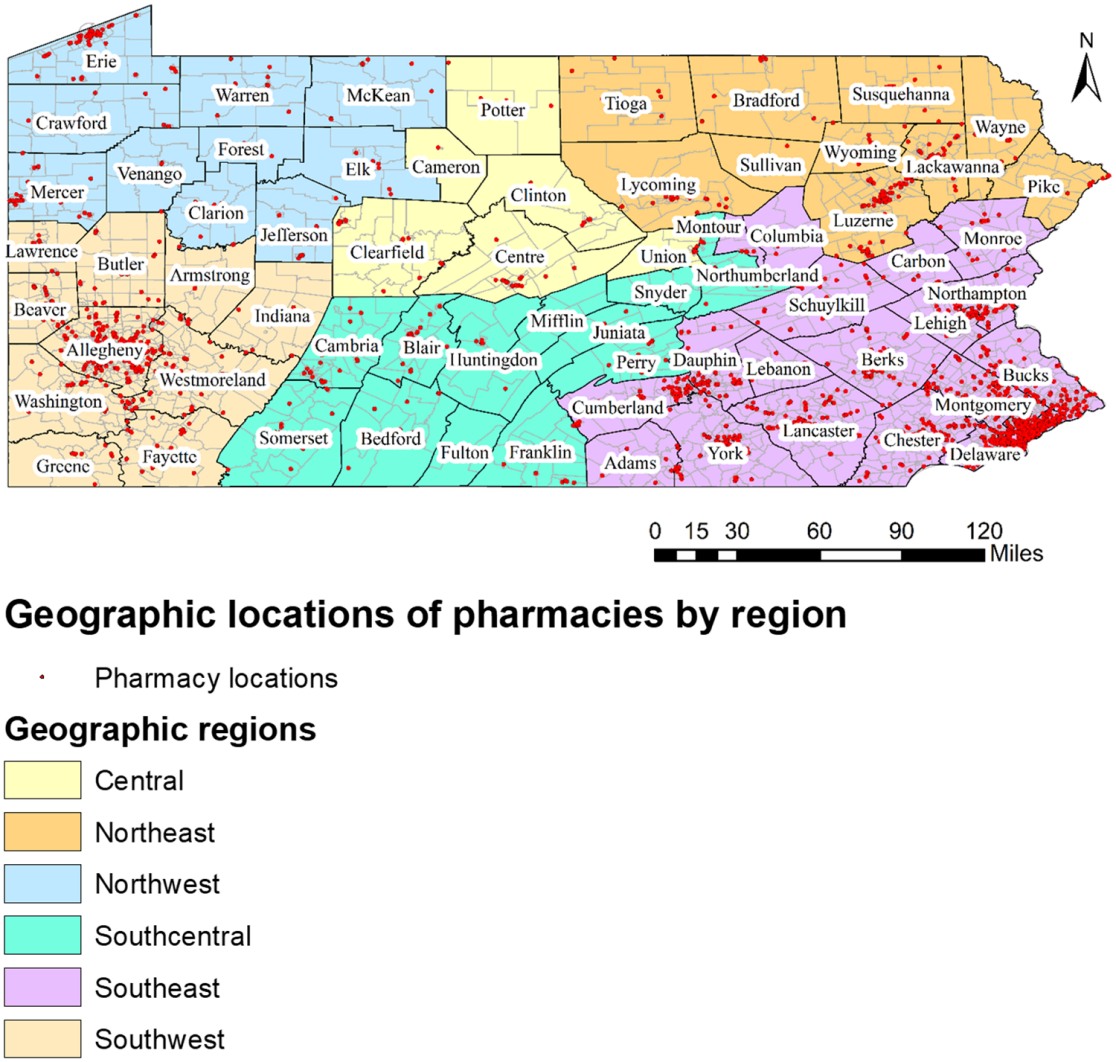
Pharmacy locations in Pennsylvania by geographic regions, 2015.

Fig 1 shows the distribution of all community pharmacies which participated in PACE program in 2015 by geographic regions in Pennsylvania. The visual depiction shows that the clusters of pharmacies were prominent in Southeast and Southwest regions of Pennsylvania.

The distance to the nearest community pharmacy was calculated for a total of 172,967 enrollees living in 3,013 of the 3,218 total census tracts in Pennsylvania. Of these, 1,183 (39%) census tracts, which included 69,555 enrollees, were identified as pharmacy deserts and 1,830 (61%) census tracts, which included 103,412 enrollees, were identified as pharmacy non-deserts (p-value < 0.001). For a list of census tracts which were identified as pharmacy deserts and pharmacy non-deserts, refer to S1 File.

**Fig 2:**
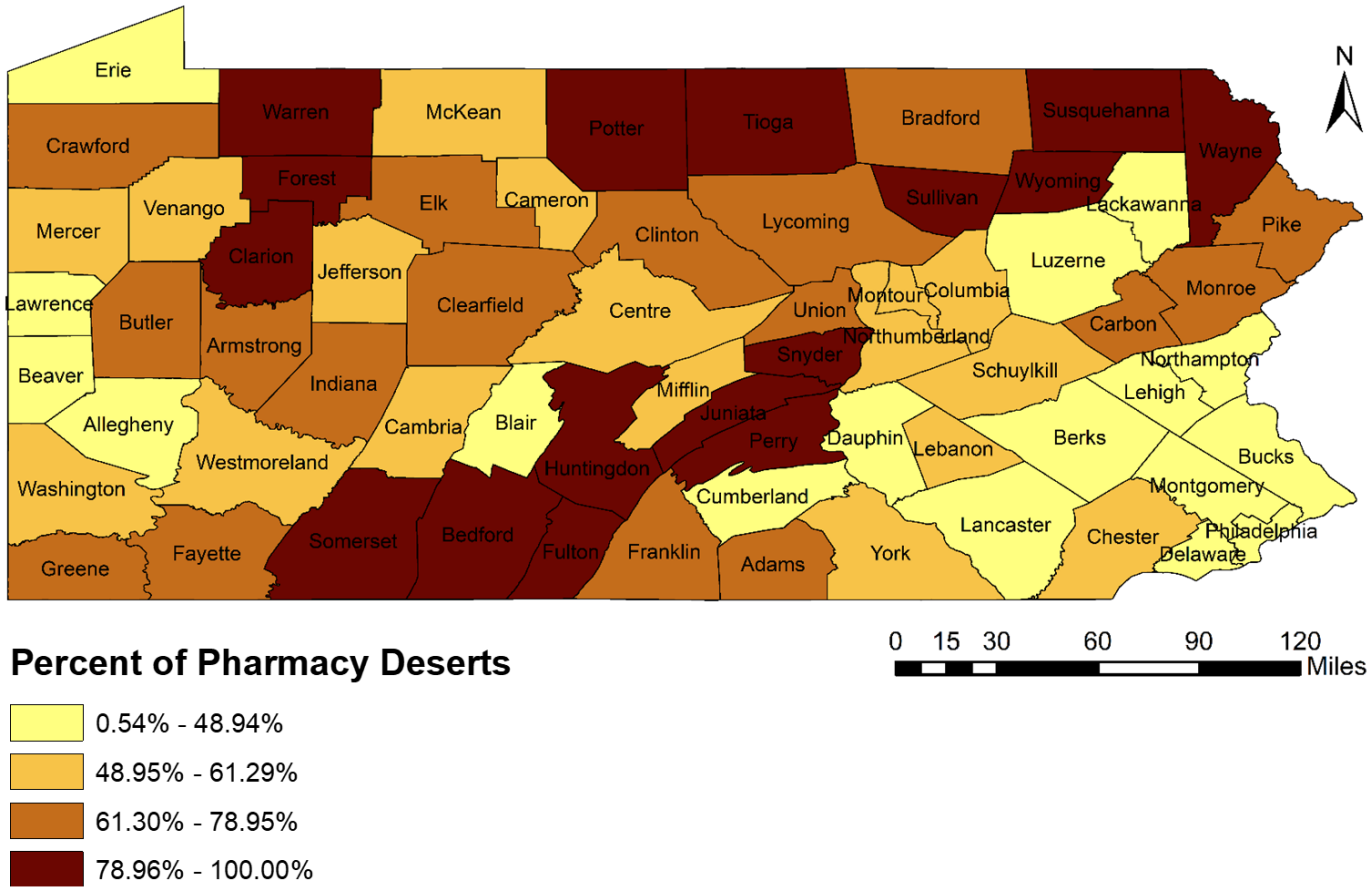
A quartile map of percent of pharmacy deserts at county level in Pennsylvania, 2015.

**Fig 3:**
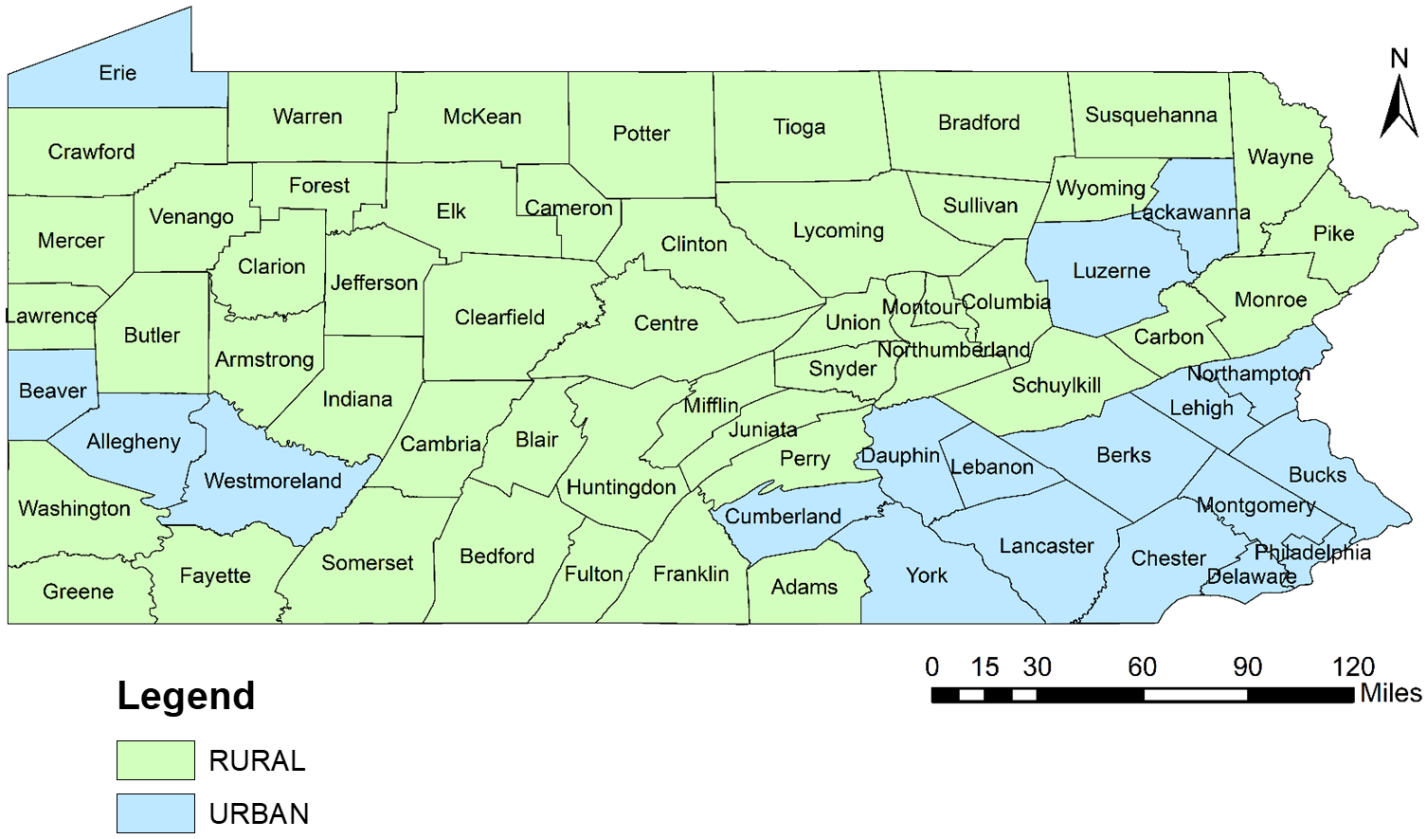
Urban and rural counties in Pennsylvania, 2015.

There are Total 67 counties in Pennsylvania. Each county had at least one pharmacy desert (which was defined at census tract level). The percent of pharmacy deserts at county level in the state, which represents the geographic accessibility of pharmacies, is shown visually in Fig 2. It illustrates that pharmacy deserts were more prevalent among counties located in Southcentral (Somerset, Bedford, Fulton, Huntingdon, Juniata, Perry, Snyder), Northeast (Tioga, Sullivan, Susquehanna, Wyoming and Wayne), Central (Potter) and Northwest (Warren, Forest and Clarion) regions in Pennsylvania. Though pharmacy deserts were comparatively less within counties in Southeast and Southwest regions, there were total 15 counties (Carbon, Monroe, Schuylkill, Columbia, Montour, Lebanon, York, Chester, Butler, Armstrong, Indiana, Washington, Greene, Westmoreland, Fayette), which had more than 50% census tracts identified as pharmacy deserts. Fig 3 shows the distribution of urban and rural areas at county level in Pennsylvania. Comparison of Figs 2 and 3 indicates that the pharmacy deserts are more prevalent in rural counties. More details specific at county level are provided in Supporting Information Files section [see S2 File].

**Fig 4:**
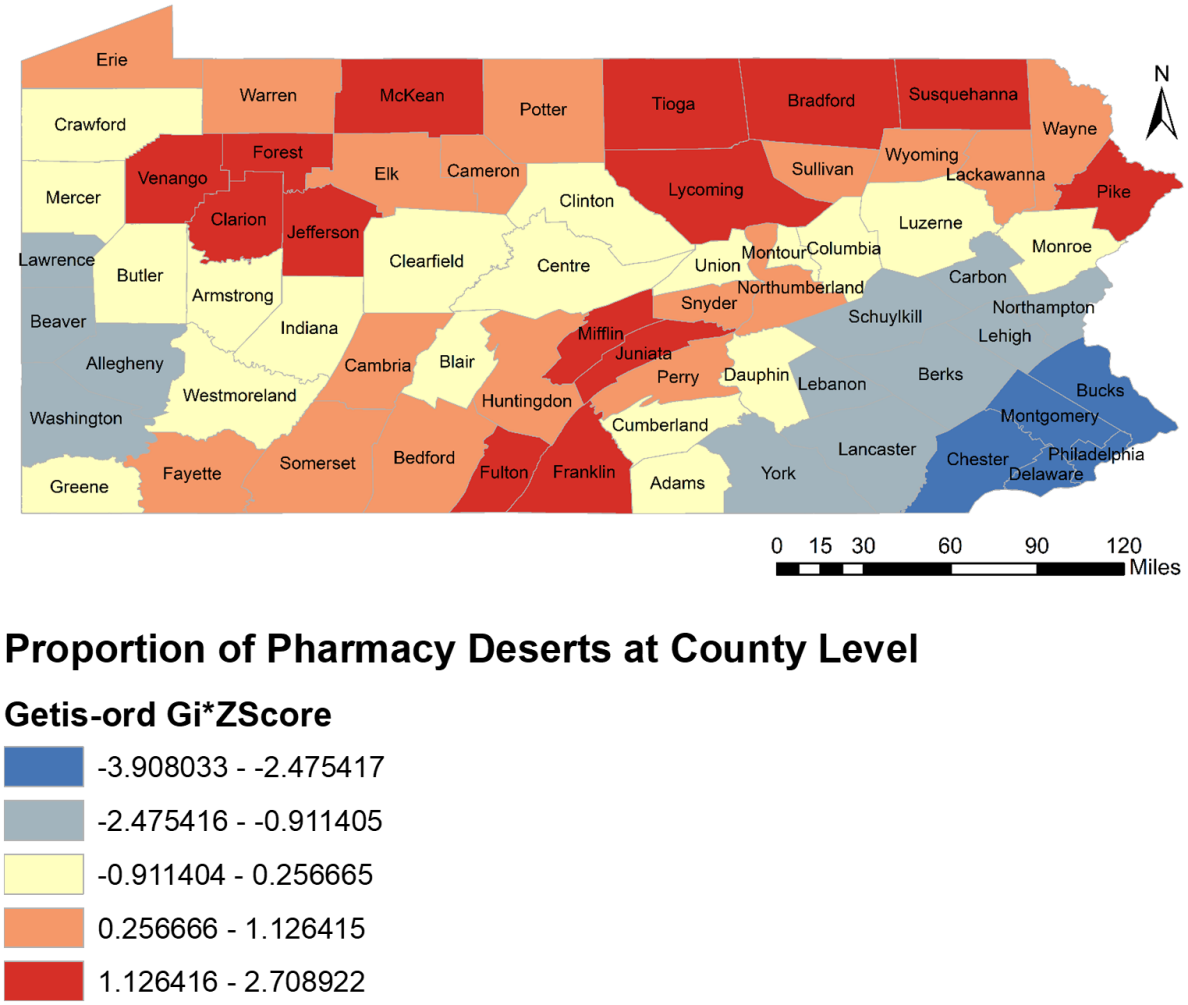
Hot spot analysis of pharmacy deserts at the county level in Pennsylvania, 2015.

Results from hot spot analysis at the county level portray a detailed picture of the statistically significant clusters of pharmacy deserts (Fig 4). In statewide analyses (67 counties), we found statistically significant hot spot clusters (Red shading) in 31 counties, with the highest proportion of pharmacy deserts (dark red) having a mean PPD of 0.74 ± 0.18 for 14 counties; and the light red with an average PPD of 0.72 ± 0.24 for 17 counties. Significant cold spot clusters (blue shading) existed in 17 counties, with the lowest PPD (dark blue) in 5 counties (mean PPD = 0.25 ± 0.23); and light blue with a mean PPD of 0.45 ± 0.15 for 12 counties. Total 19 (28.4% of 67) counties (yellow colored) had statistically non-significant PPD with mean PPD of 0.57 ± 0.16.

**Fig 5:**
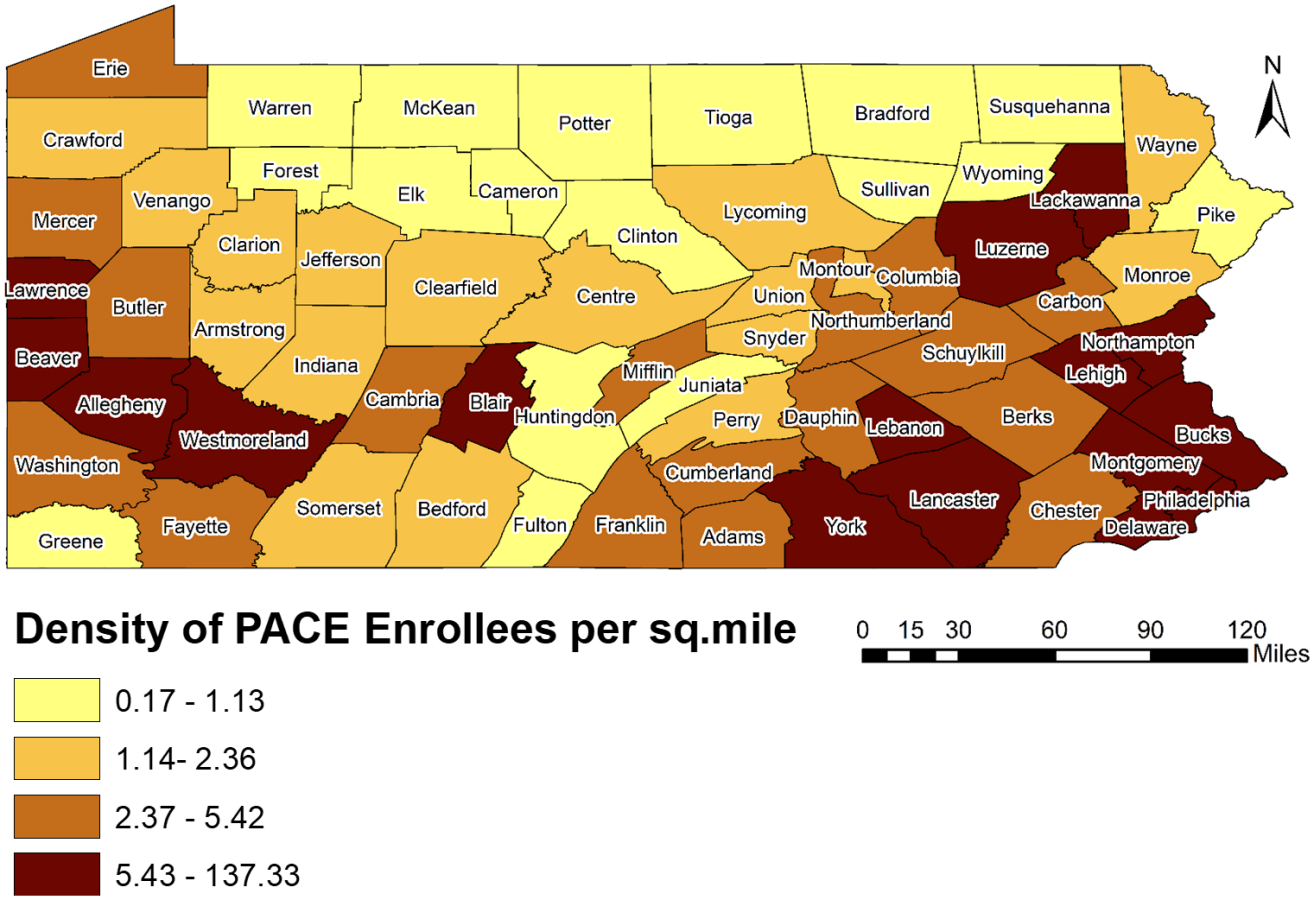
Density of PACE enrollees in Pennsylvania by county, 2015.

Fig 4 also indicates that hot spots (with higher PPD in a county) tended to be in the northern counties of the state, while the southern part of the state had a mix of hot (higher PPD) and cold (lower PPD) spots. The greatest concentration of cold spots was in the southeast and southwest region of the state. These regions represent many of the urban areas of the state containing higher density of PACE enrollees (Figs 3 and 5). The hot spots were more concentrated in southcentral, northwest and northeast regions of the state. The counties included in the hot spots were rural areas containing lower density of PACE enrollees (Figs 3 and 5). However, Lackawanna and Erie counties were the exceptional hot spots as they were urban areas with higher density of PACE enrollees.

### II. Socioeconomic inequalities, racial/ethnic minority hot spots and pharmacy deserts

Table 2 provides the selected socio-economic and demographic characteristics of the sampled enrollees living in identified pharmacy deserts and pharmacy non-deserts in Pennsylvania. The population was predominantly female (71%) and most enrollees were white (84%). Enrollees living in pharmacy deserts were slightly younger than enrollees living in pharmacy non-deserts (78.5 years vs. 78.8 years, p < 0.0001). Median annual household income was significantly higher for enrollees living in pharmacy deserts than those living in pharmacy non-deserts ($17,517 vs. $16,223 p <0.0001). Pharmacy deserts were more likely to have males (31%), married (33%), white (93%) and non-Hispanic (99%) enrollees as compared to pharmacy non-deserts.

**Table 2:** Socio-economic and demographic characteristics of the sampled enrollees by community type, 2015.

Variable	Level	Total		Pharmacy Deserts		Pharmacy Non-deserts		p-value
		N	%	N	%	N	%	
**Total number of enrollees**	-	172,967	-	69,555	-	103,412	-	-
**Population density per square mile**	-	15.05	-	1.72^a^	-	28.38^b^	-	-
**Age at enrollment in PACE (in years)**	Mean (S.D)	78.73	(± 7.58)	78.50	(± 7.40)	78.88	(± 7.69)	<0.0001^1^
**Annual household income (in USD)**	Median (IQR)	16,715	(7,632)	17,517	(8,170)	16,223	(7,259)	<0.0001^1^
**Sex**	Male	49,631	28.7	21,804	31.4	27,827	26.91	<0.0001^2^
	Female	123,336	71.3	47,751	68.6	75,585	73.09	
**Marital Status**	Single/Widowed	105,846	61.2	38,980	56.0	66,866	64.7	<0.0001^2^
	Married	44,609	25.8	23,064	33.2	21,545	20.8	
	Divorced	18,896	10.8	6,370	9.2	12,226	11.8	
	Married but living separately	3,916	2.3	1,141	1.6	2,775	2.7	
**Race**	White	145,177	83.9	64,532	92.8	80,645	77.9	<0.0001^2^
	African American	13,072	7.6	704	1.0	12,368	11.9	
	Other^3^	1,943	1.12	416	0.2	1,527	1.5	
	Multiple	777	0.5	197	0.3	580	0.6	
	Unknown	11,998	6.9	3,706	5.3	8,292	8.0	
**Ethnicity**	Hispanic	2,964	1.7	511	0.7	2,453	2.4	<0.0001^2^
	Non-Hispanic	170,003	98.3	69,044	99.3	100,959	97.6	

^1^T-test was statistically significant at α = 0.05

^2^Chi-square test was statistically significant at α = 0.05

^3^Includes American Indian and Alaska Native, Asian, Pacific Islander and other

^a^Total land area of census tracts defined as pharmacy deserts = 40,461.4 sq. miles

^b^Total land area of census tracts defined as pharmacy non-deserts = 3,644.2 sq. miles

**Fig 6:**
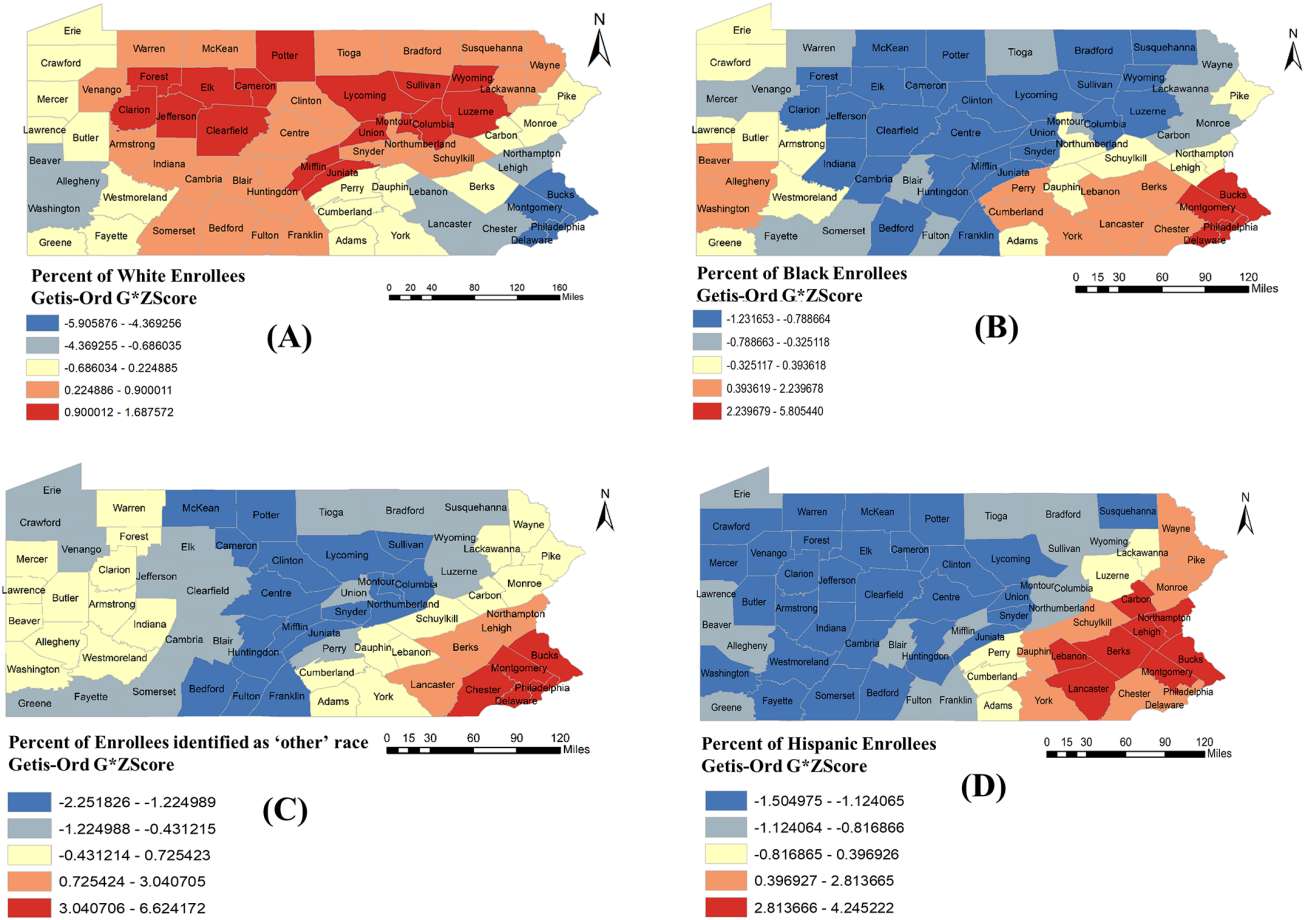
County-specific hot spot maps for the percentage of PACE enrollees by racial-ethnic group in Pennsylvania, 2015: (A) White; (B) Black; (C) ‘Other’ including Asian, American Indian and Alaska Native, Pacific Islander and other; (D) Hispanic.

The results of hot spot analyses (Fig 6) showed that African American hot spots (red counties representing higher percentage of black enrollees) were mainly concentrated in Southeast and Southwest regions (Fig 6B). On the contrary, white cold spots (blue counties representing lower percentage of white population) were found to be in those regions (Fig 6A). Enrollees who were identified as ‘Other’ race were concentrated in east part of Southeast region of Pennsylvania (Fig 6C). Hispanic hot spots were also concentrated in Southeast region of the state (Fig 6D). Based on the maps (Figs 4 and 6), it is evident that pharmacy deserts are less prevalent in minority communities and more concentrated in counties with higher percentage of white population.

### III. Pharmacy deserts and accessibility to community pharmacies and their services

There were total 3,195 pharmacies in Pennsylvania which participated in the PACE program in 2015. After excluding pharmacies which were not independent or chain pharmacies, we found that there were 2,752 pharmacies in Pennsylvania, of which only 24% pharmacies were present in pharmacy deserts (Table 3).

**Table 3:** Access to community pharmacies in pharmacy deserts and pharmacy non-deserts in Pennsylvania, 2015.

Characteristics of Community Pharmacies		Total	Pharmacy Deserts	Pharmacy Non-deserts	p-value
**Total number of community pharmacies**, n (%)		2,752	648 (24)	2,104 (76)	-
**Total pharmacy density per 100 enrollees** (mean ± S.D)		3.33 ± 14.67	1.76 ± 6.32	4.34 ± 18.06	<0.0001^1^
**Density of Independent pharmacy per 100 enrollees** (mean ± S.D)		1.16 ± 6.74	0.59 ± 3.92	1.53 ± 8.04	<0.0001^1^
**Density of Chain pharmacy per 100 enrollees** (mean ± S.D)		2.16 ± 11.25	1.17 ± 4.81	2.80 ± 13.87	<0.0001^1^
**Delivery Service**, n (%) (Missing values = 443)	Yes	965 (34.9)	184 (28.3)	781 (37.0)	<0.0001^2^
	No	1,355 (49.0)	362 (55.7)	993 (46.9)	
**Emergency Delivery Service**, n (%) (Missing values = 442)	Yes	1,243 (45.0)	291 (44.8)	952 (45.1)	0.8489
	No	1,078 (39.0)	256 (39.4)	822 (38.9)	
**Everyday Service**, n (%) (Missing values = 445)	Yes	1,385 (50.1)	337 (51.8)	1,048 (49.6)	0.2826
	No	933 (33.8)	209 (32.2)	724 (34.3)	
**24-Hours Service** (Missing values = 450)	Yes	32 (1.2)	2 (0.3)	30 (1.4)	0.0192^2^
	No	2,281 (82.6)	543 (83.5)	1,738 (82.3)	
**Total number of enrollees per pharmacy** (n)		63	107	49	-
**Distance to nearest pharmacy in miles**, median (range)		0.59 (0.29–1.55)	2.09 (1.08–3.92)	0.36 (0.20–0.58)	0.0001^1^

^1^T-test was statistically significant at α = 0.05

^2^Chi-square test was statistically significant at α = 0.05

**Fig 7:**
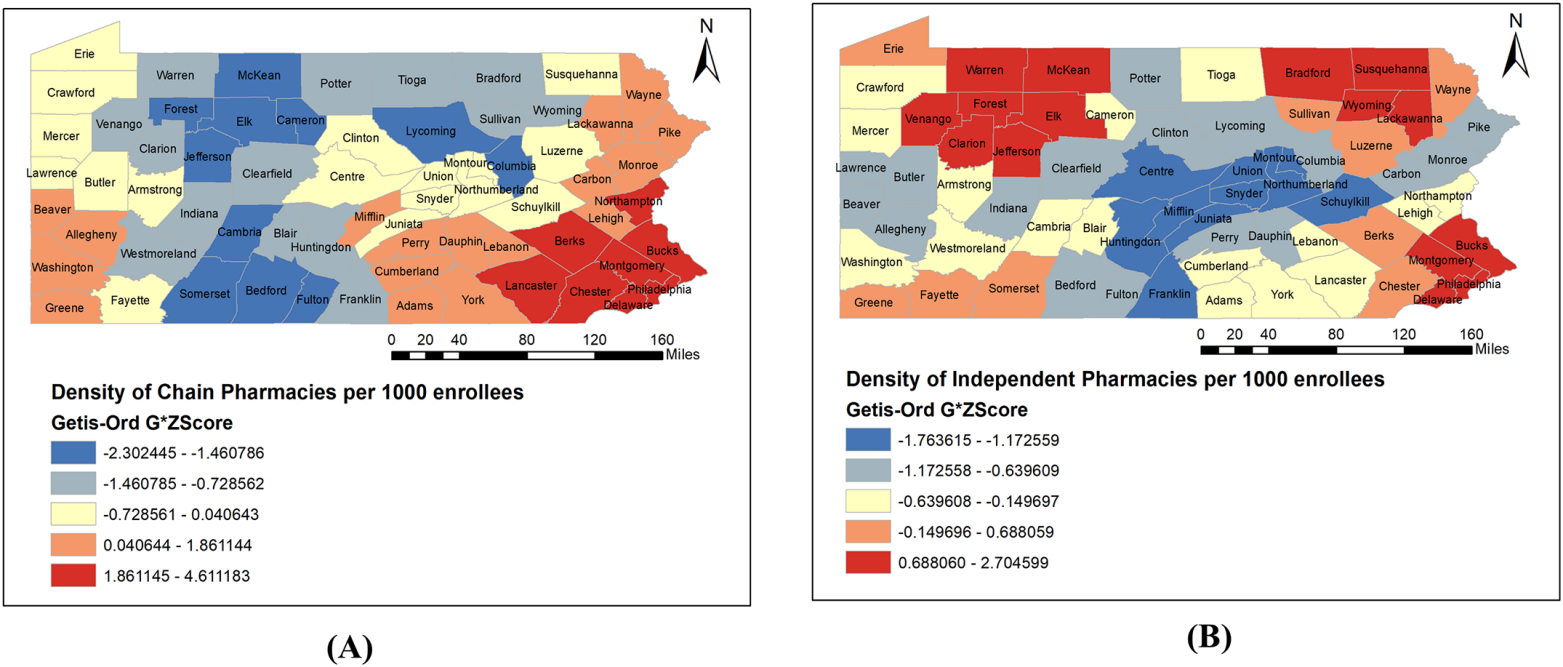
County-specific hot spot maps for density of (A) chain and (B) independent pharmacies per 1000 PACE enrollees in Pennsylvania, 2015.

Table 3 describes the characteristics of community pharmacies and services present in identified pharmacy deserts and pharmacy non-deserts in the state of Pennsylvania. Independent and chain pharmacies were more likely to be found in the pharmacy non-deserts than pharmacy deserts (p < 0.0001). Fig 7 depicts the statistically significant clusters of different types of community pharmacies at county level. Hot spot clusters of chain pharmacies (Fig 7A) were found to be in Southeast and Southwest regions, exactly where pharmacy non-deserts are more prevalent (Fig 4). Cold spots of chain pharmacies were concentrated in the regions including counties with high proportion of pharmacy deserts (Figs 4 and 7A). Hot spot clusters of independent pharmacies were prevalent among counties with both more PPD (Northeast and Northwest regions) as well as less PPD (Southeast region) (Figs 4 and 7B). For more details about the accessibility to pharmacies in each census tract is provided in S1 File.

From Table 3 Pharmacy deserts were less likely to have delivery services (28.3% vs. 37.0%, p <0.0001) and 24-hours services (1.2% vs. 0.3%, p = 0.0192) than pharmacy non-deserts. However, there was no significant difference in the presence of emergency delivery services and everyday services offered in pharmacies located in pharmacy deserts and pharmacy non-deserts.

We found that each pharmacy located in pharmacy deserts had to provide service to more enrollees than a pharmacy located in pharmacy non-deserts (107 vs. 49, respectively). There was a significant difference in distance to nearest pharmacy between enrollees residing in pharmacy deserts and pharmacy non-deserts (p = <0.0001), with enrollees living in pharmacy deserts having to travel more to visit the nearest pharmacy as compared to those living in pharmacy non-deserts (median = 2.09 miles vs. 0.36 miles respectively). For the mean distance to nearest community pharmacy for each census tract in Pennsylvania, see S1 File.

## Discussion

The results of these analyses identified pharmacy deserts and investigated availability of different types of community pharmacies and their services, that can affect geographic access to medications and other services offered by community pharmacies for elderly even if their economic access was improved by being enrolled in SPAPs. The study findings confirm the presence of spatial heterogeneity in data. The results can be used to inform interventions aimed at improving access to prescription medications and community pharmacies and SPAP program management by targeting specific geographic areas which need more attention, rather than delivering interventions to the entire study area. The results can also be used as a guide for resource allocation decisions, target outreach efforts, and help guide public health policy and program enhancement decisions. The methodological approach and analyses demonstrated here for PACE program can also be applied to other public health programs in the US and globally to evaluate the coverage and breadth of public health services. Several states in countries around the world, such as New Zealand, Brazil, and Canada, are currently experiencing similar problems associated with geographic accessibility to community pharmacies[20,28,50].

This study detected clusters of pharmacy deserts at county level in the southcentral, northwest and northeast regions of the state. These clusters of high proportion of PPD appeared to parallel areas of low densities of PACE enrollees, while the clusters of low PPD appeared to overlap with areas of high densities of PACE enrollees. County-level hot spot analysis highlighted locations in which the PACE directors could consider outreaching to more community pharmacies to participate in the SPAP, or the state government could consider providing incentives to increase the number of community pharmacies on the local level. Conversely, statistically significant cold spots highlighted regions in which expanded PACE services or more number of pharmacies may not have been needed and in which PACE may have been adequately serving those communities’ needs. Further research needs to be done to investigate whether the coverage of PACE services and number of community pharmacies are adequate to serve neighborhood’ needs in pharmacy deserts and non-deserts.

The clusters of pharmacy deserts were located in rural areas and pharmacy non-deserts were more prevalent in urban areas. These findings are consistent with the findings of the previous studies that residents of rural areas generally have to travel more to visit a pharmacy[24]. Pharmacy deserts were found to have fewer blacks and Hispanics and more white individuals compared to pharmacy non-deserts. Hot spot analysis also indicated that the counties with lower PPD mostly overlapped with counties with less percentage of PACE enrollees of racial and ethnic minority groups. These findings are contradictory to results found in other studies, which have demonstrated that the racial and ethnic minority communities generally have low access to pharmacies and other community services as compared to whites[19,26]. This can be explained by the fact that PACE enrollees belonging to racial/ethnic minority groups were predominantly located in urban areas where pharmacy deserts were found to be less.

Pharmacy deserts had less total pharmacy density and less chain pharmacy density per 100 enrollees than pharmacy non-deserts. This finding is consistent with past studies which have shown that pharmacy owners, particularly chain pharmacies, generally make decisions about market entry and exit based on the population density and income status of the community [51,52]. Hot spot analysis also showed that clusters of high density of chain pharmacies were located in pharmacy non-deserts, which are urban areas. The results can be used to target interventions at these geographic areas to attract more chain pharmacies to participate in the PACE program. Pharmacy deserts had fewer independent pharmacies as compared to pharmacy non-deserts per 100 enrollees. This may be explained by the idea that independent pharmacies may have closed their business in pharmacy deserts due to competition with chain pharmacies and low population density. Hot spot maps, however, revealed that clusters of higher density of independent pharmacies were located in both urban and rural counties. Further research is needed to study whether distribution of different types of pharmacies present in pharmacy deserts and pharmacy non-deserts is parallel with the communities’ needs. More delivery services in pharmacy non-deserts than pharmacy deserts can be explained by the higher number of independent pharmacies which generally provide such services to thrive in competition with chain pharmacies. Fewer 24-hour pharmacies in pharmacy deserts, despite having more chain pharmacies than non-deserts, can be explained by the low population density. However, the results of distribution of pharmacy services across pharmacy deserts and non-deserts should be analyzed with caution considering the missing data.

Our study only included elderly enrolled in PACE and examined their accessibility to pharmacies in PACE network. As our study did not involve out-of-network pharmacies, where PACE enrollees are not allowed to fill their prescriptions through SPAP, possible *edge effect* has been removed. It is important to note some limitations of the study. This study has used the distance calculated based on the haversine formula which considers spherical shape of the earth to identify the nearest pharmacy and did not measure the driving or walking distance (over the road networks) people travel. Hence, it is possible that when the road network and transportation means available in the city are considered, the nearest pharmacy identified by our method may be more inconvenient or even farther. The future research should consider total network travel distance and time while identifying geographic accessibility to a community pharmacy. Future researchers should consider the means of travel such as foot, public transit, private vehicle and driving distance while calculating network distance or time. It is also possible that study participants could have moved to a different address from what was recorded in the dataset later in the year. We also assumed that patients refilled their prescriptions only at the pharmacy nearest to their residences. Though it is a logical assumption, we cannot be completely certain that it is true. Patients may choose a pharmacy which is located on their journey, where medications are available, or based on the referrals and past experiences with the pharmacy services. Market dynamics including competition between supplies and availability of frequently prescribed medications may also affect patient’s accessibility to medications even though a pharmacy is located nearby patient’s residence. Future researchers should consider these factors while developing the criteria for pharmacy deserts. Patients may refill their prescriptions in pharmacies located in hospitals, nursing homes, physician offices or long-term care facilities, however, they were not considered in this study. Since this study focused on access to community pharmacies used by elderly residents, we did not consider other avenues of acquiring prescription medications, such as via internet, mail-order or through family member or social network member.

Our study extends findings from previous research that focused on access to health care and to medications for elderly, a vulnerable population. Knowing the geographic locations of “hot spots” of pharmacy deserts is critical to improve the geographic accessibility to pharmacies in the communities and help in increasing intervention efficiency. Future research investigating the effect of pharmacy deserts and availability of different types of community pharmacies and pharmacy services on the health outcomes such as medication adherence rates, healthcare costs and utilization is highly warranted.

## Conclusions

To the best of our knowledge, this study is the first to report pharmacy deserts and availability of community pharmacies and their services for elderly enrolled in a SPAP and our methodological approach should be reasonably generalizable to other public health programs globally.

The findings of our study suggest that geographic access to a nearby community pharmacy, the type of pharmacy and pharmacy services vary significantly across pharmacy deserts and pharmacy non-deserts. This study shows the need for improvements in the distribution of pharmaceutical services in rural areas. The findings of this study also showed that racial and ethnic disparities exist in geographic accessibility to a nearby pharmacy for elderly. Moreover, additional studies are required to better understand the relationship between pharmacy deserts and healthcare utilization and costs to develop effective strategies to achieve equitable access to medications and pharmacy services in disadvantaged communities.

## Supporting information

S1 File Sociodemographic characteristics and geographic accessibility to a community pharmacy for the census tracts in Pennsylvania, 2015. S1 File contains details at Census Tracts level of Pennsylvania for 2015 in terms of County name in which it is located, geographic information such as land area per sq. mile, pharmacy desert status and mean distance to nearest pharmacy; socio-demographic details of PACE enrollees living in the Census Tract; and information about different types of community pharmacies.
(XLSX)

S2 File Sociodemographic characteristics and geographic accessibility to a community pharmacy for counties in Pennsylvania, 2015. S2 File contains details at County level of Pennsylvania for 2015 in terms of geographic region in which it is located, other geographic information such as land area per sq. mile, urban-rural designation, percent of pharmacy deserts and mean distance to nearest pharmacy; socio-demographic details of PACE enrollees living in the County; and information about different types of community pharmacies.
(XLSX)
